# Perceived antisemitism and the mental health of Jewish university students in Germany: a quantitative comparative study

**DOI:** 10.1186/s12889-026-27714-5

**Published:** 2026-05-22

**Authors:** Tal Josef Tamir, Irina Jarvers

**Affiliations:** 1 Sigmund Freud Privat University– Berlin, Berlin, Germany; 2 Department of Child and Adolescent Psychiatry and Psychotherapy, University of Regensburg, Regensburg, Germany

**Keywords:** Jewish students, Antisemitism, Minority stress, Mental health; University students

## Abstract

**Background:**

Antisemitism has increased in Germany in recent years, raising concerns about its impact on the mental health of Jewish university students. However, empirical research on mental health outcomes among Jewish students in Germany remains limited.

**Methods:**

This cross-sectional study used a quantitative comparative design to examine stress, anxiety, depression, and self-esteem among Jewish and non-Jewish university students. An online survey was completed by 320 students, including 151 who identified as Jewish. Measures assessed perceived antisemitism, Jewish identification, self-esteem, and connection to Israel. Group differences were analyzed, and predictors of mental health outcomes were examined within the Jewish subsample.

**Results:**

Jewish students reported significantly higher levels of stress, anxiety, and depression than their non-Jewish peers, while no significant differences were observed in self-esteem. Within the Jewish subsample, perceived antisemitism emerged as the strongest predictor of psychological distress, whereas self-esteem functioned as a protective factor. Stronger connection to Israel was associated with elevated stress.

**Conclusions:**

The findings indicate that perceived antisemitism represents a significant mental health risk factor for Jewish university students in Germany. Addressing minority stress and strengthening protective resources within higher education settings may help reduce psychological distress and promote student well-being.

**Supplementary Information:**

The online version contains supplementary material available at 10.1186/s12889-026-27714-5.

## Introduction

The Hamas terrorist attack on Israel on October 7, 2023, and the subsequent war in Gaza coincided with a sharp rise in antisemitism in Germany. Antisemitic incidents increased by nearly 83% in 2023, with more than half occurring after the attack and many involving violent or threatening expressions [5]. In 2024, 8,627 antisemitic incidents were documented, compared with 4,886 in 2023, representing an additional increase of nearly 77% [6]. The German Federal Ministry of the Interior likewise reported record levels of antisemitic crime [4]. This escalation, referred to here as "October 7th and its aftermath," has significantly affected the sense of safety within Jewish communities.

In response, some Jews have increased security measures, limited public expressions of Jewish identity, or considered emigration [5]. However, the psychological consequences of these developments, particularly for Jewish university students navigating both campus life and heightened societal tensions, remain underexplored. This gap is especially salient in Germany, where antisemitism unfolds within a post-Holocaust memory culture and strong institutional commitments to combating antisemitism, yet public Jewish visibility continues to entail vulnerability (Federal Government Commissioner for Jewish Life in Germany and the Fight against Antisemitism 13.

University students represent a population already vulnerable to stress, anxiety, and depression, particularly during transitional periods [1, 22]. These challenges are often exacerbated by cultural and minority-related stressors [21]. In Germany, a recent meta-analysis found that 21.1% of higher education students report depressive symptoms [17], and more than half reported worsening mental health during the COVID-19 pandemic [15]. For Jewish students in particular, university campuses have emerged as hotspots for antisemitism: following October 7th, exposure to hate and fear increased significantly, and many no longer felt comfortable expressing their cultural identity on campus [33]. Antisemitism-related stress has been independently linked to heightened depressive symptoms in this population, with coping strategies playing a key moderating role [27].

In the present study, antisemitism is understood in accordance with the International Holocaust Remembrance Alliance [18] working definition, as a perception of Jews that may be expressed through hostility, prejudice, or discrimination toward Jewish individuals, communities, or institutions. To examine the psychological consequences of such experiences, the present study applies Minority Stress Theory [25, 26], which conceptualizes mental health disparities as the result of chronic, socially rooted stressors linked to marginalized identities. Within this framework, antisemitism can function as a key minority stressor for Jewish students.

Such stress may operate not only through direct experiences but also through perceived hostility, expectations of rejection, and heightened vigilance. Within a minority stress framework, antisemitism can be understood as a chronic, identity-based psychosocial stressor [26]. Building on this conceptualization, recent work has developed dedicated measures to assess antisemitism-related stress within Jewish populations [31]. Research consistently links both experienced and perceived antisemitism to increased anxiety, depression, and reduced well-being [24, 35, 38], mirroring broader findings on perceived discrimination [9, 34].

Jewish identity is a multidimensional construct encompassing religious, cultural, and national components [14, 20]. These dimensions reflect different ways in which individuals may experience belonging to the Jewish people, including religious practice, cultural affiliation, and connections to Israel. In the present study, *identity* refers to these substantive dimensions of belonging, whereas *identification* denotes the subjective strength with which individuals relate to their Jewish identity. Accordingly, the religious dimension is operationalized through measures of religiosity and religious identification, the cultural dimension through cultural identification, and the national dimension through connection to Israel.

In the German context, Jewish identity is often not outwardly visible, and many Jews actively manage this visibility in response to perceived threat [2]. Prior research suggests that identity can function both as a protective resource and a source of vulnerability: while strong identification may foster resilience, it may also increase visibility and thus exposure to discrimination, or may heighten vigilance towards antisemitism [31, 35]. Connection to Israel represents a particularly salient dimension, serving as a source of identity while also constituting a potential stressor in politicized environments, especially during periods of conflict [10, 11].

Despite extensive research on student mental health and minority stress, Jewish students in Germany remain an understudied group. Existing studies primarily focus on the United States or Israel, older populations, or pre–October 7th contexts. Addressing this gap, the present study examines stress, anxiety, depression, and self-esteem among Jewish university students in Germany, with particular attention to perceived antisemitism, dimensions of Jewish identity, connection to Israel, and self-esteem as a potential protective factor. A comparative component with non-Jewish students situates these findings within broader student mental health patterns. In the present study, perceived antisemitism refers to participants’ perceptions of the prevalence and severity of antisemitic attitudes across different social domains, including antisemitism in German society, Israel-related antisemitism, and antisemitism within academic environments.

In addition to identity-related factors, selected sociodemographic variables were included based on prior research linking contextual and vulnerability-related characteristics to mental health disparities. Sexual orientation was considered in light of Minority Stress Theory, as LGBTQ + individuals face compounded minority stressors that may intersect with other identity-based sources of discrimination and elevate risk for psychological distress [26]. Political orientation has been associated with differences in perceived socio-political threat and stress reactivity [36], and urban upbringing has been linked to increased exposure to chronic environmental stressors and higher risk of affective disorders [19]. A history of psychiatric conditions was included as an established vulnerability factor for subsequent stress and depression [16].

Taken together, these theoretical and empirical considerations suggest that both identity-related factors and broader sociodemographic characteristics may shape the psychological impact of antisemitism on Jewish students. Specifically, Jewish students are expected to report higher psychological distress than non-Jewish peers due to their additional exposure to identity-based minority stressors, including perceived antisemitism and the associated processes of vigilance and expectation of rejection. Minority Stress Theory provides an integrative framework linking exposure to antisemitism and related identity-based stressors to elevated psychological distress [9, 26, 34], while personal resources such as self-esteem may moderate the extent to which these stressors translate into adverse mental health outcomes.

The study pursues three aims: (1) to assess differences in mental health outcomes between Jewish and non-Jewish students; (2) to examine associations between identity-related factors and mental health among Jewish students; and (3) to identify predictors of psychological distress within both the full sample and the Jewish subgroup. We hypothesized that Jewish students would report higher levels of stress, anxiety, and depression than their non-Jewish peers, and that perceived antisemitism and connection to Israel would be associated with greater psychological distress, while self-esteem and aspects of Jewish identification would function as protective factors.

## Methods

### Participants

A total of 371 individuals accessed the online survey, recruited through convenience-based snowball sampling. Eligible participants were university students residing in Germany who self-identified as Jewish or non-Jewish. Recruitment channels included Jewish student organizations, posters on Berlin campuses, and Jewish and Israeli social media networks (e.g., Facebook, WhatsApp, Signal). Data collection took place between June 25 and September 2, 2024. After excluding 46 non-students and 5 non-residents, the final sample comprised 320 university students (Mean age = 26.6, *SD* = 5.60), of whom 61.9% identified as female and 47.2% as Jewish (*n* = 151).

The majority held German citizenship (73.6%), while 12.8% were Israeli nationals and the rest had mixed citizenship. In the total sample, political orientation was predominantly left-leaning (86.9%, *M* = 2.92, *SD* = 1.96), urban-raised (55%), and enrolled in bachelor’s programs (55.6%). A majority reported a personal (59.9%) or familial (56.6%) psychiatric history. Within the Jewish subsample, 71.5% had a history of migration, 55.6% were born in Germany, and 58.3% of those with a history of migration were native German speakers.

While the sampling approach enabled access to a diverse student population, it may have introduced selection bias, with overrepresentation of students from Berlin (56.6%) and those from the social sciences (33.4%) in the total sample.

An a priori power analysis using G*Power [12] indicated that a sample of at least 109 participants would be sufficient to detect medium-sized effects (f2 = 0.15) in multiple regression models with eight predictors (α = 0.05, power = 0.80).

### Measures

All participants completed a demographic questionnaire followed by standardized mental health assessments. Jewish participants additionally answered identity-specific items. The online survey combined (a) previously published standardized instruments (AJIS, HADS, PSS-10, RSES), (b) items adapted from previously published surveys [10, 39], and (c) additional items developed by the authors for this study to assess perceived antisemitism, perceived safety, and connection to Israel. The newly developed items assessed perceived antisemitism, perceived safety, connection to Israel, and selected identity- and experience-related variables. The full wording of all author-developed study-specific items is provided in Supplementary Material S1.

### Demographic variables

Participants reported age, gender, nationality, income, sexual orientation, relationship status, study level and field, place of upbringing, political orientation, and psychiatric history (self and family). Political orientation was assessed using a single-item self-placement scale ranging from 0 (Far Left) to 10 (Far Right), allowing for nuanced positioning along the political spectrum. Sample characteristics across groups are presented in Table 1.

**Table 1:** Sociodemographic Characteristics, American Jewish Identity Scales Descriptive Statistics, and Mental Health Outcome Scores of the Participants

Categorical Variables	Jewish (*n* = 151, 47.2%)	Non-Jewish (*n* = 169, 52.8%)	Total (*n* = 320, 100%)	χ²	*p*	φ	
Gender (*N*, *%*)							
Male	53 (35.1%)	51 (30.2%)	104 (32.5%)	1.21	0.55	0.06	
Female	91 (60.3%)	107 (63.3%)	198 (61.9%)				
Diverse	7 (4.6%)	11 (6.5%)	18 (5.6%)				
Sexual Orientation (*N*, *%*)							
Heterosexual	92 (60.9%)	82 (48.5%)	174 (54.4%)	5.03	0.13	0.81	
Bi- and Pansexual	44 (29.1%)	63 (37.3%)	107 (33.4%)				
Queer	15 (9.9%)	24 (14.2%)	39 (12.2%)				
Place of Upbringing (*N*, *%*)							
Rural area	14 (9.3%)	25 (14.8%)	39 (12.2%)	11.54	0.009	0.19	
Town	14 (9.3%)	33 (19.5%)	47 (14.7%)				
Medium-sized city	27 (17.9%)	31 (18.3%)	58 (18.1%)				
Large city	96 (63.6%)	80 (47.3%)	176 (55%)				
AJIS (*M*, *SD*)							
Cultural Scale	2.85 (0.71)	-	-	-	-	-	
Religious Scale	2.01 (0.82)						
Total Scale	2.43 (0.68)						

**Continuous Variables**	**Jewish** **(*n* = 151, 47.2%)**	**Non-Jewish** **(*n* = 169, 52.8%)**	**Total** **(*n* = 320, 100%)**	**Mann–Whitney U**	**Z**	**p (2-tailed)**	**r**
Age (*M*,* SD*)	26.58 (6.01)	26.64 (5.21)	26.61 (5.59)	12207.00	-0.67	0.503	0.04
Political Orientation (*M*,* SD*)	3.45(1.97)	2.43 (1.81)	2.92 (1.96)	8602.50	-5.13	< 0.001	0.29
Anxiety (HADS)	Range =	Range =	Range =	9189.00	-4.33	< 0.001	0.24
Normal (0–7) = 19.4%	0.0–21.0	0.0–21.0	0.0–21.0				
Borderline (8–10) = 25.3%	M = 12.05	M = 10.05	*M* = 10.99				
Probable clinical (≥ 11) = 55.3%	SD = 4.23	SD = 4.07	*SD* = 4.23				
Depression (HADS)	Range =	Range =	Range =	10,558.00	-2.67	0.007	0.15
Normal (0–7) = 63.7%	0.0–16.0	0.0–18.0	0.0–18.0				
Borderline (8–10) = 24.1%	M = 6.70	M = 5.76	*M* = 6.20				
Probable clinical (≥ 11) = 12.2%	SD = 3.55	SD = 3.90	*SD* = 3.76				
Perceived Stress (PSS)	Range =	Range =	Range=	10,803.00	-2.37	0.018	0.13
low (0–13) = 10.0%	8.0–38.0	3.0–40.0	3.0–40.0				
moderate (14–26) = 61.9%	M = 23.48	M = 21.62	*M* = 22.5				
high (27–40) = 28.1%	SD = 6.19	SD = 6.88	*SD* = 6.62				
Self-Esteem (RSES)	Range =	Range =	*Range = *	11,745.00	-1.23	0.219	0.07
	10.0-40.0	14.0–40.0	*10.0–40.0*				
	M = 27.72	M = 26.97	*M = 27.32*				
	SD = 5.95	SD = 5.66	*SD* = 5.8				

Note. AJIS = American Jewish Identity Scales; HADS = Hospital Anxiety and Depression Scale, PSS = Perceived Stress Scale, RSES = Rosenberg Self-Esteem Scale. Categories for place of upbringing were defined according to population size: large city (> 100,000), medium-sized city (20,000–100,000), town (5,000–20,000), rural area (< 5,000 inhabitants)

Jewish students answered items adapted from the EU Fundamental Rights Agency [10, 39], along with additional demographic/background questions (country of birth, years in Germany, history of migration, German language proficiency). In addition, new items were developed, assessing perceived antisemitism, perceived safety, and connection to Israel (see Supplementary Material S1 for the English-language item wording).

*Jewish identity* was assessed with four indicators: (1) strength of identification (11-point scale), (2) religiosity (11-point scale), (3) frequency of community involvement, and (4) self-categorization (predefined and open-ended).

*Perceived antisemitism* was measured with four items: (1) estimated percentage of Germans holding antisemitic views, and perceived levels of (2) general, (3) Israel-related, and (4) academic antisemitism. The latter three items were combined into a composite score demonstrating good internal consistency (α = 0.80); inter-item correlations ranged from ρ = 0.50 to ρ = 0.57 (all *p* < 0.001), supporting the coherence of the scale.

*Perceived safety* was measured with two items: (1) general safety as a Jew in Germany and (2) the impact of the Arab–Israeli conflict on feelings of safety as a Jewish person in Germany. The term "Arab–Israeli conflict" reflects the original wording of the survey item and was intended as a broad geopolitical reference rather than to convey a particular political framing.

*Connection to Israel* was assessed with five items covering personal/family ties, identity relevance, and news engagement. Items were combined into a composite variable (α = 0.70).

### Jewish identity scale

A shortened version of the American Jewish Identity Scales (AJIS) [14] was used to reduce survey length. Five cultural and five religious items were selected based on their strong psychometric properties in the original validation study, preserving the scale’s two-dimensional structure. Items were rated on a 4-point Likert scale. Internal consistency for the subscales was high (α = 0.76–0.88). The scale captures general dimensions of cultural (e.g., "I am proud to be Jewish") and religious (e.g., "I observe the Sabbath") identification, which are not tied to specific national contexts.

### Mental health outcomes

Anxiety and depression were assessed with the Hospital Anxiety and Depression Scale (HADS) [40]. Perceived stress was measured with the 10-item Perceived Stress Scale (PSS-10) [7]. Self-esteem was measured using the Rosenberg Self-Esteem Scale (RSES) [32]. All scales demonstrated good internal consistency (α ≥ 0.80).

Although the study includes a broad set of sociodemographic and identity-related variables, some potentially relevant predictors (such as academic performance, social support, or campus climate) were not assessed. These omissions reflect necessary limits on survey length and participant burden. Nonetheless, the selected variables align with Minority Stress Theory and the study’s aims, ensuring that the analytical models remain theoretically coherent despite this constraint.

### Procedure

The study was hosted on Microsoft Forms, a European Union’s General Data Protection Regulation (GDPR) compliant platform. The survey was administered in English, as the sample included Israeli participants with limited German proficiency; this also ensured accessibility for participants with diverse linguistic backgrounds and allowed us to use the original validated versions of the instruments, thereby avoiding potential translation-related bias. After completing an informed consent form detailing study objectives, data handling, and withdrawal rights, participants proceeded to the survey.

The study was approved by the Ethics Commission of the Faculty for Psychotherapy Science, Psychology, and Law of the Sigmund Freud Private University, Vienna.

Average completion time was 12.5 min. Data were collected anonymously and stored securely on Microsoft 365 servers, with restricted access. No data were shared with third parties.

### Data analysis

Data were analyzed using SPSS (Version 29.0.2.0). All questionnaire items were set as mandatory in Microsoft Forms, preventing item-level missing data. Prior to analysis, the dataset was screened for outliers and inconsistencies.

State exam students were categorized as postgraduates for consistency. Dual citizenship was categorized by primary national affiliation. Country of birth entries (e.g., "former USSR") were retained as originally entered. Income was recorded as either parental or personal, depending on availability, to ensure one income value per respondent. These steps supported clearer group-level comparisons.

### Statistical analysis

Prior to analysis, the normality of all continuous variables was assessed using the Shapiro–Wilk test and visual inspection of histograms and Q-Q plots. As the majority of variables significantly violated the normality assumption, non-parametric tests were employed throughout. To assess the robustness of the findings, sensitivity analyses were conducted using parametric alternatives alongside the primary non-parametric tests. All analyses were conducted in SPSS Version 29.0.2.0, and statistical significance was set at α = 0.05.

#### Group differences in mental health outcomes

The data analysis followed a stepwise approach beginning with group comparisons. Mann–Whitney U tests were employed to examine differences in stress, anxiety, depression, and self-esteem between Jewish and non-Jewish students.

#### Associations between identity-related variables and mental health

Correlational analyses were conducted within the Jewish subsample to assess the relationships between identity-related factors (e.g., religiosity, perceived antisemitism, connection to Israel) and mental health outcomes. Spearman’s rho was used for continuous variables, including perceived antisemitism, connection to Israel, religious identification, cultural identification, religiosity, community involvement, and self-esteem, while Mann–Whitney U and Kruskal–Wallis tests were applied for categorical predictors including gender, sexual orientation, and place of upbringing.

#### Regression models predicting mental health outcomes

Multiple linear regression analyses were conducted to identify predictors of stress, anxiety, and depression across the full sample and the Jewish subgroup. For the full sample models, predictors included age, gender, place of upbringing, sexual orientation, political orientation, income, personal and familial psychiatric history, and Jewish identification. For the Jewish subsample models, predictors included the above sociodemographic variables that were significant alongside history of migration, perceived antisemitism, connection to Israel, religiosity, religious identification, and self-esteem.

## Results

### Descriptive analysis

#### Levels of identification within sample of Jewish students

Jewish participants reported a strong sense of Jewish identification (*M* = 8.19, *SD* = 1.97, on a 0–10 scale), with most indicating that their identification had significantly increased over the past year (*M* = 0.87, *SD* = 0.88 on a − 2 to + 2 scale, *t*(150) = 12.18, *p* < 0.001, *r* = 0.71).

When asked to describe their Jewish identity, most participants identified as secular (29.1%), “just Jewish” (23.2%), or Reform/Liberal (15.9%). Smaller proportions described themselves as Traditional (18.5%), Orthodox (6.6%), Mixed (4.6%), Masorti/Conservative (1.3%), or Haredi (0.7%), reflecting a predominantly non-Orthodox, culturally oriented sample.

On a 10-point religiosity scale, participants rated themselves as relatively non-religious (*M* = 3.26, *SD* = 2.59).

Cultural identification was moderate (*M* = 2.85, *SD* = 0.71), while religious identification was lower (*M* = 2.01, *SD* = 0.82), according to the AJIS (*z* = −9.47, *p* < 0.001). The overall AJIS score averaged 2.43 (*SD* = 0.68; see Table 1).

Community involvement varied: 22.5% reported no involvement, 31.8% reported some involvement, and the remainder reported moderate to high involvement, suggesting an even distribution of engagement in Jewish organizations.

#### Perceptions of antisemitism and feelings of safety in Germany

**Fig. 1:**
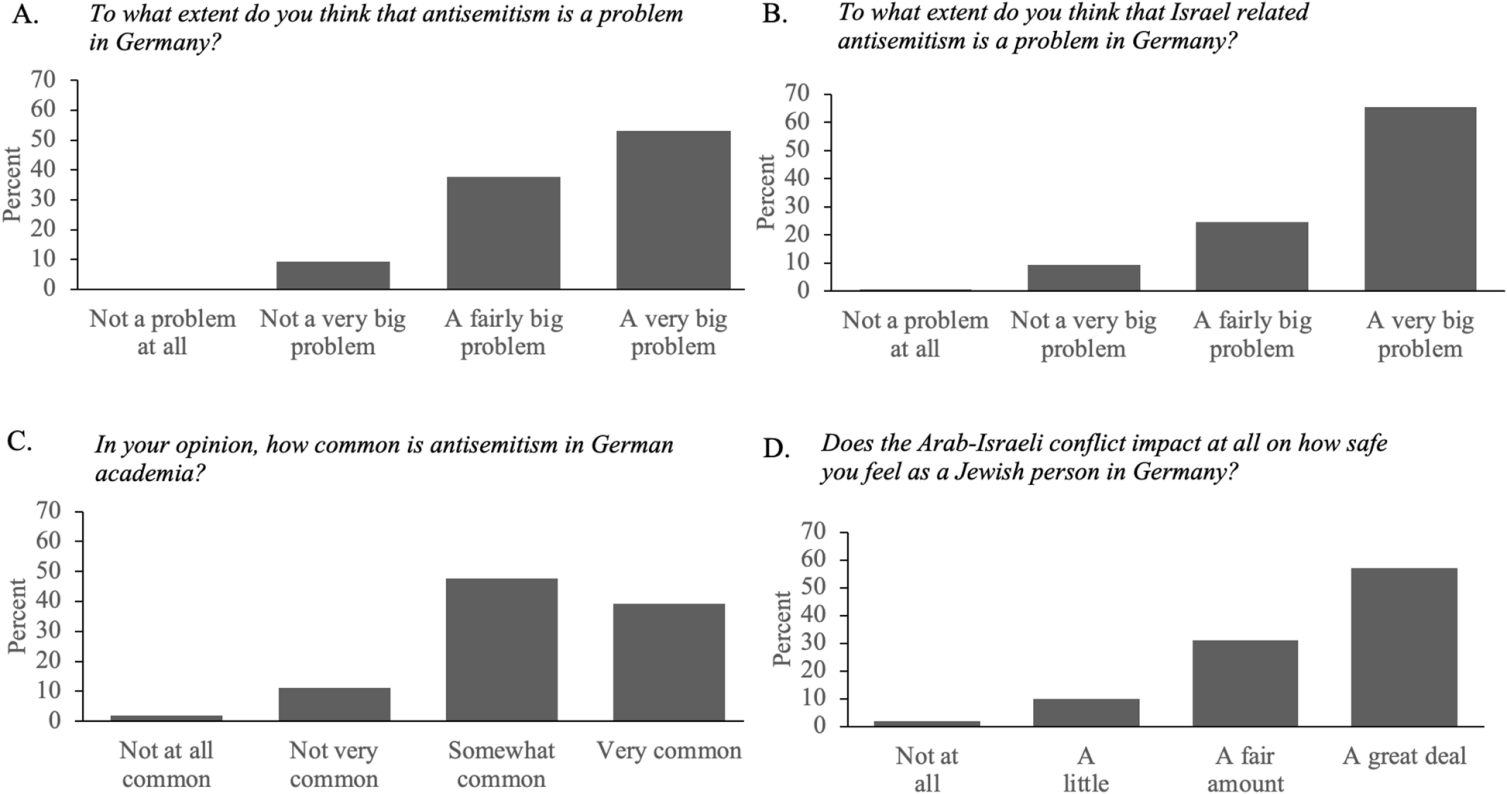
Perceptions of antisemitism and safety among Jewish individuals in Germany. **A** Extent to which antisemitism is considered a problem in Germany, **B** extent Israel related antisemitism is a problem in Germany, **C** how common antisemitism is in Germany, and **D** whether the Arab–Israeli conflict impacts how safe the individual feels as a Jewish person in Germany

Perceptions of antisemitism and safety among Jewish students were also examined. Participants estimated that, on average, 46.5% of the German population holds negative views of Jews (*SD* = 23.8). Most considered antisemitism in Germany a “very big” or “fairly big” problem, with even greater concern for Israel-related antisemitism and antisemitism in academia (see Fig. 1A–C). The combined index across these domains reflected a high level of concern (*M* = 3.41, *SD* = 0.58). Compared to a year earlier, participants perceived a significant increase in antisemitism (*M* = 1.56, *SD* = 0.73 on a − 2 to + 2 scale, *t*(150) = 26.31, *p* < 0.001, *r* = 0.91).

Feelings of safety were low: 54.3% reported feeling “not at all” or “not very” safe as Jews in Germany, while only 5.3% felt “very safe.” Most reported that the Arab–Israeli conflict had affected their sense of safety “a great deal” or “a fair amount” (Fig. 1D), illustrating the broader impact of international events on perceived vulnerability in Germany.

#### Relationship to Israel

Following this, we explored participants’ emotional and familial connection to Israel. Among Jewish participants, Israel’s importance was widely affirmed: 80.1% of participants viewed its existence as “very important,” and over 92% considered it at least moderately important. For 76.9%, Israel played a meaningful role in their Jewish identity, with 41.1% describing it as a “very big part.” The mean score across five related items was 2.96 (*SD* = 0.58), reflecting a moderate to strong connection with Israel.

Most participants (86.1%) reported some form of direct contact with Israel: 13.9% had lived there most of their lives, 17.9% for over a year, and 57.6% had visited as tourists. Only 10.6% had never been.

Family ties were also common: 66.2% reported relatives in Israel, ranging from “some” to “most or all” of their family. About one quarter (24.5%) had no family ties or were unsure.

In terms of media engagement, 87.4% reported following Israeli news either “very closely” (47.7%) or “regularly” (39.7%).

### Dependent variables

Participants reported elevated symptoms across several mental health domains (see Table 1). On average, anxiety scores fell in the borderline-to-clinical range, while depression scores were lower overall. Stress levels were predominantly moderate, with a substantial minority reporting high stress. In contrast, self-esteem scores indicated generally positive self-evaluations across the sample.

### Inferential analysis

#### Comparison of Jewish and Non-Jewish students

A Mann–Whitney U test revealed that Jewish students had higher anxiety scores (*U* = 9189.5, *Z* = −4.333, *p* < 0.001, *r* = 0.24), higher depression scores (*U* = 10,557.5, *Z* = −2.674, *p* = 0.007, *r* = 0.15) and higher stress (*U* = 10,803.0, *Z* = −2.371, *p* = 0.018, *r* = 0.13) than non-Jewish students. There was no significant difference in self-esteem scores between the groups (*U* = 11,744.5, *Z* = −1.230, *p* = 0.219, *r* = 0.07). Results are summarized in Table 1.

#### Associations between mental health outcomes, well-being and continuous variables among Jewish students

Spearman’s rho correlations were used to examine associations between mental health outcomes (stress, anxiety, depression), self-esteem, and identity-related variables among Jewish students (see Table 2).

**Table 2:** Correlation matrix of mental health outcomes and identity-related continuous variables among Jewish students (*N* = 151)

	1	2	3	4	5	6	7	8
1. Anxiety (HADS)	1.00							
2. Depression (HADS)	.62***	1.00						
3. Perceived Stress (PSS)	.70***	.64***	1.00					
4. Self-Esteem (RSES)	-.38***	-.47***	-.55***	1.00				
5. Religious Identification	-.04	.02	-.04	-.06	1.00			
6. Perceived Antisemitism	.30***	.20*	.32***	-.05	-.08	1.00		
7. Connection to Israel	-.05	.09	-.05	.19*	-.03	.08	1.00	
8. Community Involvement	-.08	-.08	-.11	.14	.48***	-.03	-.04	1.00

^*^ *p* ≤.05, *** *p* <.001

Perceived antisemitism was significantly associated with higher levels of anxiety, depression, and stress, while self-esteem showed moderate negative correlations with all three distress outcomes. The strongest association observed was between perceived stress and self-esteem (ρ = –0.55, *p* < 0.01), suggesting a protective effect of self-worth.

Connection to Israel was weakly but significantly correlated with higher self-esteem, though not with anxiety, depression, or stress. Religious identification and community involvement were not significantly associated with any mental health variables.

#### Perceived changes in antisemitism and Jewish identity: exploratory

Participants were asked whether their perceptions of antisemitism and Jewish identity had changed over the past year and to name events they believed influenced these shifts. A large majority (86.8%) attributed the rise in antisemitism to “the 7th of October and its aftermath,” referring to the Hamas attacks, the war in Gaza, and related incidents in Germany. Other responses, such as the rise of the far right, social media, or economic instability, were mentioned only rarely.

Similarly, over half (53.0%) cited the same event as the main factor influencing changes in their Jewish identity, followed by engagement in Jewish communities (15.9%) and changes in personal or social settings. A smaller number indicated no change or could not name a specific event.

A Spearman’s rho correlation confirmed a positive association between perceived increases in antisemitism and strengthened Jewish identity (ρ = 0.27, *p* < 0.001), suggesting that rising threat perceptions may reinforce identity salience among Jewish students.

#### Multiple regression analysis predicting stress, anxiety, and depression for all students

Three multiple regression models were conducted to examine predictors of stress, anxiety, and depression across all participants (*N* = 320), including age, gender, upbringing, sexual and political orientation, income, psychiatric history, and Jewish identification (see Table 3).

**Table 3:** Multiple regression analysis predicting stress, anxiety, and depression for all students (*N* = 320) and for Jewish students (*N* = 151)

		All Students (*N* = 320)				Jewish Students (*N* = 151)			
Outcome	Predictor	B	β	t	p	B	β	t	p
Stress	Age	−0.05	−0.04	−0.73	.467	-	-	-	-
	Gender	1.26	0.12	1.90	.058	-	-	-	-
	Place of Upbringing	−0.53	−0.09	−1.66	.097	-	-	-	-
	Sexual Orientation	−0.32	−0.03	−0.61	.542	-	-	-	-
	Political Orientation	**−0.62**	**−0.18**	**−3.01**	**.003**	**−0.75**	**−0.24**	**−3.47**	** <.001**
	Income	−0.36	−0.12	−2.38	.018	−0.24	−0.09	−1.38	.171
	Psychiatric History (Personal)	**3.29**	**0.25**	**4.32**	** <.001**	1.20	0.10	1.44	.153
	Psychiatric History (Family)	−0.07	−0.03	−0.52	.603	-	-	-	-
	Jewish Identification	**2.96**	**0.22**	**4.17**	** <.001**	0.21	0.07	0.98	.338
	History of Migration	-	-	-	-	−0.14	−0.01	−0.16	.875
	Perceived Antisemitism	-	-	-	-	**2.59**	**0.24**	**3.98**	** <.001**
	Connection to Israel	-	-	-	-	**1.56**	**0.15**	**2.26**	**.026**
	AJIS Religion Scale	-	-	-	-	0.11	0.01	0.20	.840
	Self-Esteem	-	-	-	-	**−0.57**	**−0.55**	**−8.28**	** <.001**
Anxiety	Age	0.02	0.03	0.50	.621	-	-	-	-
	Gender	0.75	0.10	1.79	.074	-	-	-	-
	Place of Upbringing	−0.10	−0.02	−0.47	.637	-	-	-	-
	Sexual Orientation	0.32	0.05	0.96	.336	-	-	-	-
	Political Orientation	**−0.48**	**−0.22**	**−3.76**	** <.001**	**−0.68**	**−0.31**	**−4.03**	** <.001**
	Income	−0.14	−0.08	−1.50	.136	-	-	-	-
	Psychiatric History (Personal)	1.53	0.18	3.17	.002	−0.12	−0.01	−0.18	.856
	Psychiatric History (Family)	0.10	0.06	1.16	.248	-	-	-	-
	Jewish Identification	**2.77**	**0.33**	**6.17**	** <.001**	**0.33**	**0.15**	**1.98**	**.049**
	History of Migration	-	-	-	-	0.45	0.05	0.67	.503
	Perceived Antisemitism	-	-	-	-	**1.95**	**0.27**	**3.81**	** <.001**
	Connection to Israel	-	-	-	-	0.70	0.10	1.26	.211
	AJIS Religion Scale	-	-	-	-	0.15	0.03	0.35	.726
	Self-Esteem	-	-	-	-	**−0.28**	**−0.39**	**−5.17**	** <.001**
Depression	Age	**0.07**	**0.11**	**2.01**	**.045**	0.06	0.10	1.43	.155
	Gender	−0.14	−0.02	−0.36	.720	-	-	-	-
	Place of Upbringing	**−0.45**	**−0.13**	**−2.35**	**.019**	−0.26	−0.07	−1.02	.308
	Sexual Orientation	0.03	0.01	0.10	.923	-	-	-	-
	Political Orientation	−0.21	−0.11	−1.76	.079	-	-	-	-
	Income	−0.18	−0.11	−1.93	.055	-	-	-	-
	Psychiatric History (Personal)	0.82	0.11	1.80	.073	-	-	-	-
	Psychiatric History (Family)	0.03	0.02	0.40	.687	-	-	-	-
	Jewish Identification	**1.41**	**0.19**	**3.29**	**.001**	0.09	0.05	0.65	.516
	History of Migration	-	-	-	-	0.57	0.07	0.99	.325
	Perceived Antisemitism	-	-	-	-	**1.21**	**0.20**	**2.79**	**.006**
	Connection to Israel	-	-	-	-	0.80	0.13	1.81	.073
	AJIS Religion Scale	-	-	-	-	0.16	0.04	0.46	.647
	Self-Esteem	-	-	-	-	**−0.29**	**−0.48**	**−6.70**	** <.001**

In the full sample, “Jewish Identification” indicates group status (Jewish vs. non-Jewish). In the Jewish subsample, it refers to the strength of Jewish identity. Reference categories for categorical predictors: gender = male; sexual orientation = heterosexual; place of upbringing = rural area; personal psychiatric history = no; familial psychiatric history = no, history of migration = no. Significant variables are marked in bold

All models were statistically significant, explaining 19.6% of the variance in stress (*R*^*2*^ = 0.196), 22.3% in anxiety (*R*^*2*^ = 0.223), and 9.7% in depression (*R*^*2*^ = 0.097).

Across outcomes, Jewish identification consistently predicted higher levels of distress: stress (*β* = 0.22, *p* < 0.001), anxiety (*β* = 0.33, *p* < 0.001), and depression (*β* = 0.19, *p* = 0.001). Additional significant predictors included personal psychiatric history (stress and anxiety), lower income (stress), older age and urban upbringing (depression), and more left-leaning political orientation (stress and anxiety). No other variables reached significance.

#### Multiple regression analysis predicting stress, anxiety, and depression for Jewish students

Regression models were conducted to identify predictors of psychological distress among Jewish students, including self-esteem, history of migration, perceived antisemitism, connection to Israel, religiosity, Jewish identification, political orientation, and relevant sociodemographic variables (see Table 3).

All three models were significant (*p* < 0.001), explaining 52.0% of the variance in stress (*R*^*2*^ = 0.520), 36.1% in anxiety (*R*^*2*^ = 0.361), and 33.3% in depression (*R*^*2*^ = 0.333).

Across outcomes, perceived antisemitism was consistently associated with higher distress, while self-esteem emerged as a strong protective factor. In addition, connection to Israel predicted higher stress, and left-leaning political orientation was linked to higher stress and anxiety. Jewish identification, religiosity, and history of migration were not significant in any model.

#### Sensitivity analyses

To assess the robustness of the findings, sensitivity analyses were conducted using parametric alternatives alongside the primary non-parametric tests. Independent samples t-tests for group comparisons yielded substantively identical results to the Mann–Whitney U tests across all outcomes (anxiety: *t*(318) = 4.32, *p* < 0.001, *d* = 0.48; depression: *t*(318) = 2.24, *p* = 0.026, *d* = 0.25; stress: *t*(318) = 2.53, *p* = 0.012, *d* = 0.28; self-esteem: *t*(318) = 1.15, *p* = 0.252, *d* = 0.13). Similarly, Pearson's correlations produced the same pattern of significant and non-significant associations as Spearman's rho across all identity-related and mental health variables. These results confirm that conclusions are robust regardless of the analytic approach chosen.

## Discussion

This study examined mental health outcomes among Jewish university students in Germany in the context of heightened antisemitism following the Hamas terrorist attack on Israel on October 7, 2023. Consistent with Minority Stress Theory, Jewish students reported significantly higher levels of stress, anxiety, and depression than their non-Jewish peers [26], while self-esteem did not differ between groups. Within the Jewish subsample, perceived antisemitism emerged as the most consistent correlate of psychological distress, whereas self-esteem functioned as a protective factor. In contrast, a stronger connection to Israel was associated with elevated stress and depression, highlighting the complex and context-dependent role of identity-related factors during periods of geopolitical conflict.

### Principal findings and interpretation

The elevated levels of psychological distress observed among Jewish students underscore the mental health burden associated with minority status in a context of rising antisemitism. Although high levels of stress, anxiety, and depression are common among university students in general [1, 29], the pronounced differences between Jewish and non-Jewish students suggest an added burden linked to minority-specific stressors. These findings align with Minority Stress Theory, which posits that members of stigmatized groups experience chronic social stressors that adversely affect mental health beyond general life stress [26].

Perceived antisemitism emerged as a central driver of distress among Jewish students. This is consistent with prior research linking both direct and ambient forms of antisemitism, such as hostile discourse and derogatory assumptions, to anxiety, depression, psychosomatic symptoms, social withdrawal, and heightened vigilance [27, 35, 38]. These findings mirror broader evidence that perceived discrimination, independent of objective exposure, is robustly associated with poorer psychological outcomes across minority groups [9, 30, 34].

Minority Stress Theory provides a useful framework for interpreting these associations. The theory proposes that prejudice-related stressors activate chronic processes such as expectations of rejection, vigilance, and identity concealment, which in turn heighten stress reactivity and anxiety [26]. In university contexts where debates about Israel, diversity, and minority rights are highly visible [27, 33], these processes may be particularly salient. Such environments can activate identity-related threat processes, including vigilance and expectations of rejection [26], and increase exposure to discrimination, with well-documented consequences for psychological well-being [9, 30]. The associations observed in this study therefore reflect theoretically grounded pathways linking antisemitism to psychological distress rather than isolated correlational effects.

Self-esteem emerged as a consistent protective factor among Jewish students, mitigating stress, anxiety, and depression. This finding is consistent with vulnerability and resilience models suggesting that stable personal resources can buffer the impact of external stressors [28, 37]. Longitudinal research indicates that self-esteem tends to be relatively stable over time and less reactive to situational stressors than emotional states such as anxiety or depression [3]. The absence of group differences in self-esteem between Jewish and non-Jewish students further supports the interpretation that elevated distress among Jewish students reflects contextual stressors rather than diminished baseline self-worth.

The association between connection to Israel and increased stress highlights the dual role of national or transnational identity during periods of conflict. Previous research indicates that Jews in Europe often feel held responsible for Israeli policies, particularly during times of escalation, which may increase exposure to hostility and emotional strain [10, 11, 39]. Recent studies following October 7 have also shown that indirect exposure to war-related stressors, including media coverage and social discourse, was associated with increased psychological distress, which in turn predicted higher rates of substance use as a form of self-medication [8].

Political orientation was associated with stress and anxiety, with more left-leaning attitudes predicting higher distress. This aligns with research linking political orientation to differences in perceived socio-political threat and stress reactivity [36]. Greater political engagement during the polarized climate following October 7 may have increased exposure to conflict-related discourse. However, this finding should be interpreted cautiously given the predominantly left-leaning sample.

### Public health implications

These findings underscore the importance of recognizing antisemitism as a public health concern rather than solely a social or political issue. Perceived antisemitism functioned as a chronic stressor with measurable mental health consequences, consistent with public health frameworks that emphasize discrimination and social exclusion as key social determinants of health [9, 30, 34].

Universities represent critical intervention settings. Campus-based mental health services should be equipped to recognize and address minority stress related to antisemitism, including subtle and indirect forms. Culturally sensitive counseling and inclusive institutional policies may help mitigate identity-related distress among Jewish students [35]. Universities can also play a preventive role by addressing antisemitism within diversity initiatives and promoting respectful dialogue in politically charged contexts [11].

The protective role of self-esteem highlights potential intervention targets. Programs aimed at strengthening self-worth and coping skills may help buffer the mental health impact of minority stress, consistent with evidence linking self-esteem to resilience, anxiety and psychological well-being [23, 37].

### Strengths and limitations

This study has several strengths, including a relatively large sample of Jewish university students, the inclusion of a non-Jewish comparison group, and the examination of multiple mental health outcomes alongside identity-related predictors. These features allowed for the identification of both group-level disparities and within-group variation.

However, several limitations should be noted. The cross-sectional design precludes causal inference, and it remains possible that psychological distress influences perceptions of antisemitism rather than the reverse. Longitudinal research is therefore needed to clarify temporal relationships and examine mental health trajectories under changing social conditions.

The sample was not nationally representative and was skewed toward younger, urban, and predominantly secular Jewish students, limiting generalizability to more religious or geographically diverse populations. The recruitment relied on convenience-based snowball sampling, with an overrepresentation of Berlin-based and predominantly left-leaning students. While Berlin hosts the largest Jewish community in Germany, self-selection bias remains possible, as students more engaged in Jewish networks or more concerned about antisemitism may have been more likely to participate. Accordingly, the findings should be interpreted as reflecting patterns within this sample rather than nationally representative estimates for all Jewish students in Germany.

All measures were self-reported, introducing potential bias through recall or social desirability. Participant eligibility, including student status, residence in Germany, and Jewish or non-Jewish identification, was also based on self-report and could not be independently verified. In addition, some instruments were developed in non-German contexts and may not fully capture the historical and cultural specificity of Jewish life in Germany. Furthermore, while German students show generally high English proficiency, administering the survey in English may have introduced language-related response bias, particularly among participants with lower English proficiency. Several study-specific items assessing perceived antisemitism, perceived safety, and connection to Israel were author-developed or adapted from existing surveys without formal psychometric validation; reliability coefficients for composite scores are reported, but construct validity was not independently assessed. Furthermore, the limited variability in religiosity restricted the analysis of religious identification as a protective or risk factor. Additionally, history of migration was assessed as a binary variable (yes/no), and no information was collected on generational status. As first- and second-generation migrants may differ in their experiences of discrimination and identity formation, future research should assess this distinction more precisely. Finally, although Shapiro–Wilk tests indicated significant departures from normality, this test is known to be overly sensitive at the sample sizes used in the present study. Visual inspection revealed that while some outcome variables were approximately normally distributed, depression scores showed meaningful positive skew with a floor effect, supporting the use of non-parametric tests as the primary analytic approach. Sensitivity analyses using parametric alternatives confirmed the robustness of all findings.

### Future research and conclusion

Future research should employ longitudinal and cross-national designs to examine how antisemitism, identity, and mental health interact over time and across social contexts. Intervention studies are also needed to evaluate the effectiveness of strategies aimed at reducing minority stress and strengthening protective factors such as self-esteem.

## Conclusion

Jewish university students in Germany experience significantly higher levels of stress, anxiety, and depression than their non-Jewish peers in the context of rising antisemitism. Perceived antisemitism emerged as a central determinant of psychological distress, while self-esteem functioned as a protective factor. A stronger connection to Israel was associated with increased stress, highlighting the context-dependent nature of identity-related factors during periods of geopolitical conflict. These findings underscore antisemitism as a public health concern and point to universities as key settings for intervention. Targeted, culturally sensitive mental health support and institutional strategies addressing minority stress are essential to promote equity and well-being in higher education.

## Supplementary Information

Supplementary Material 1.

## Data Availability

The datasets generated and/or analysed during the current study are not publicly available due to ethical and privacy considerations but are available from the corresponding author on reasonable request.
