# Supplementary material for: Perceived antisemitism and the mental health of Jewish university students in Germany: a quantitative comparative study
Source: BMC Public Health. 2026 May 22;26:1702. doi: 10.1186/s12889-026-27714-5 (PMC13200457; doi:10.1186/s12889-026-27714-5)
Supplement: Supplementary file 1 — Supplementary Material 1. [file 12889_2026_27714_MOESM1_ESM.docx]

## **Supplementary Material S1:** **Study-specific survey items**

**Overview**This supplementary material provides the full wording of all survey items that were developed by the authors specifically for this study. Previously published standardized instruments (American Jewish Identity Scales [AJIS], Hospital Anxiety and Depression Scale [HADS], Perceived Stress Scale [PSS-10], Rosenberg Self-Esteem Scale [RSES]) and items that were directly adapted from previously published surveys (EU Fundamental Rights Agency, 2018; Zick et al., 2017) are cited in the main manuscript and are not reproduced verbatim here.

**A. Demographic and Background Variables**

1. **Are you a student?**☐ Yes
   ☐ No
2. **Do you live in Germany?**☐ Yes
   ☐ No
3. **In which federal state (Bundesland) do you live?**☐ Berlin
   ☐ Bayern (Bavaria)
   ☐ Niedersachsen (Lower Saxony)
   ☐ Baden-Württemberg
   ☐ Rheinland-Pfalz (Rhineland-Palatinate)
   ☐ Sachsen (Saxony)
   ☐ Thüringen (Thuringia)
   ☐ Hessen
   ☐ Nordrhein-Westfalen (North Rhine-Westphalia)
   ☐ Sachsen-Anhalt (Saxony-Anhalt)
   ☐ Brandenburg
   ☐ Mecklenburg-Vorpommern
   ☐ Hamburg
   ☐ Schleswig-Holstein
   ☐ Saarland
   ☐ Bremen
4. **What is your age?**(Open numeric response)
5. **What is your relationship status?**☐ Single
   ☐ In a relationship
   ☐ Married
   ☐ Divorced
   ☐ Widowed
   ☐ Other
6. **Where did you spend most of your time until you were 18 years old?**☐ Large city (more than 100,000 inhabitants)
   ☐ Medium-sized city (20,000–100,000 inhabitants)
   ☐ Small town (5,000–20,000 inhabitants)
   ☐ Rural area (fewer than 5,000 inhabitants)
7. **What is your current level of study?**☐ Bachelor’s degree
   ☐ Master’s degree
   ☐ PhD
   ☐ Other
8. **What is your field of study?**☐ Humanities
   ☐ Social sciences
   ☐ Natural sciences
   ☐ Engineering and technology
   ☐ Health sciences
   ☐ Arts
   ☐ Business and management
   ☐ Other
9. **What gender do you identify with?**☐ Woman
   ☐ Man
   ☐ Non-binary
   ☐ Other
10. **How would you describe your sexual orientation?**☐ Heterosexual
    ☐ Homosexual
    ☐ Bisexual
    ☐ Pansexual
    ☐ Asexual
    ☐ Other
11. **Political orientation***On a scale from 0 (far left) to 10 (far right), where would you place your political views?*
    (Scale: 0–10)
12. **Parental household income (monthly net income):**☐ Under €450
    ☐ €750–<€1,500
    ☐ €1,500–<€2,000
    ☐ €2,000–<€2,500
    ☐ €2,500–<€3,000
    ☐ €3,000–<€4,000
    ☐ €4,000–<€5,000
    ☐ €5,000 or more
    ☐ I don’t know
13. **Your own household income (monthly net income):**☐ Under €450
    ☐ €750–<€1,500
    ☐ €1,500–<€2,000
    ☐ €2,000–<€2,500
    ☐ €2,500–<€3,000
    ☐ €3,000–<€4,000
    ☐ €4,000–<€5,000
    ☐ €5,000 or more
14. **Do you have a history of psychiatric or mental health conditions?**☐ Yes
    ☐ No
15. **Does anyone in your immediate family have a history of psychiatric or mental health conditions?**☐ Yes
    ☐ No
    ☐ Don’t know
16. **What is your nationality? (Select all that apply)**☐ German
    ☐ Russian
    ☐ Israeli
    ☐ Ukrainian
    ☐ Other

**B. Jewish Background and Identity**

1. **In which country were you born?**☐ Germany
   ☐ Former Soviet Union
   ☐ Israel
   ☐ Other
2. **Do you have an immigration background?**☐ Yes
   ☐ No
3. **How would you describe your German language proficiency?**☐ A1
   ☐ A2
   ☐ B1
   ☐ B2
   ☐ C1
   ☐ C2
   ☐ Native German speaker
4. **How many years have you lived in Germany?**(Open numeric response)
5. **How strongly do you identify with being Jewish?**(Scale: 0–10)
6. **Compared to one year ago, how would you describe the strength of your Jewish identification today?**☐ Much stronger
   ☐ Somewhat stronger
   ☐ Remained the same
   ☐ Somewhat weaker
   ☐ Much weaker
7. **Can you name an event that you think influenced this change?**(Open-ended response)
8. **To what extent are you involved in a local Jewish community or organization?**☐ Not involved at all
   ☐ Somewhat involved
   ☐ Moderately involved
   ☐ Very involved

**C. Perceived Antisemitism**

1. **In your opinion, what percentage of people in German society have a negative view of Jews?**(Numeric response: 1–100)
2. **To what extent do you think antisemitism is a problem in Germany?**☐ A very big problem
   ☐ A fairly big problem
   ☐ Not a very big problem
   ☐ Not a problem at all
3. **To what extent do you think Israel-related antisemitism is a problem in Germany?**☐ A very big problem
   ☐ A fairly big problem
   ☐ Not a very big problem
   ☐ Not a problem at all
4. **In your opinion, how common is antisemitism in German academia?**☐ Very common
   ☐ Somewhat common
   ☐ Not very common
   ☐ Not at all common
5. **Compared to one year ago, how would you describe the level of antisemitism in Germany today?**☐ Much stronger
   ☐ Somewhat stronger
   ☐ Remained the same
   ☐ Somewhat weaker
   ☐ Much weaker
6. **Can you name an event that you think influenced this change?
   (Open-ended response)**

**D. Connection to Israel**

1. **To what extent is the existence of Israel important to you?**☐ Not important
   ☐ Somewhat important
   ☐ Moderately important
   ☐ Very important
2. **How closely do you follow news and events related to Israel?**☐ Very closely
   ☐ Regularly
   ☐ Occasionally
   ☐ Not at all

**E. Visibility and Intergenerational Experience**

1. **Have your parents or grandparents experienced persecution or discrimination because of their Jewish identity?**☐ Yes
   ☐ No
   ☐ Other
2. **Do people often assume you are not Jewish when they first meet you?**☐ Yes
   ☐ No
   ☐ Other
